# miR‐9 Restricts Insulin Secretion by Targeting Rab34, Which Mediates Lysosomal Degradation of Proinsulin

**DOI:** 10.1002/kjm2.70202

**Published:** 2026-03-27

**Authors:** Zhen‐Zhen Guo, Ao‐Ying Li, Ting‐Ting Xiang, Zu‐Hao Zhang, Ao Deng, Yang Han

**Affiliations:** ^1^ Hunan University of Humanities Science and Technology Loudi China; ^2^ The Yangzhou School of Clinical Medicine of Dalian Medical University Yangzhou China; ^3^ National Engineering Research Center for Nanotechnology Shanghai China; ^4^ Jinshan Branch of Shanghai Sixth People's Hospital Shanghai China; ^5^ Xupu Hospital of TCM Huaihua China; ^6^ Tangshan Normal University Tangshan China

**Keywords:** diabetes, insulin secretion, miRNA‐9, proinsulin, Rab34

## Abstract

Insulin secretion is a complex, vesicular transport process. Rab34 is a key regulator of intracellular vesicle transport; however, its role in insulin secretion has not yet been reported. miRNA‐9 is vital for the development and progression of the diagnosis and treatment of type 2 diabetes. This study aimed to investigate whether miR‐9 targets Rab34 to regulate insulin secretion in beta (β) cells and its molecular mechanism. We used miR‐9 mimics, miR‐9 inhibitors, and dual‐luciferase reporter gene detection to prove that miR‐9 is regulated by targeting Rab34 in Min6 cells. Transmission electron microscopy revealed a decrease in the number of insulin particles when Rab34 was overexpressed in β cells. Regarding the mechanism, Rab34 overexpression induced a decrease in the number of proinsulin‐containing particles in β cells and significantly promoted proinsulin degradation. Immunofluorescence microscopy revealed that Rab34 overexpression resulted in insulin granule clustering. Conversely, Rab34 depletion maintained proinsulin and increased insulin secretion. miR‐9 overexpression inhibited insulin secretion, and miR‐9 downregulation promoted insulin secretion. Immunofluorescence results revealed that Rab34 induced insulin secretory vesicles toward the autophagic degradation pathway. Therefore, we concluded that miR‐9 inhibits insulin secretion by promoting Rab34 expression, which regulates insulin secretion by mediating the degradation of proinsulin and autophagic degradation pathways in β cells.

## 1 Introduction

Type 2 diabetes mellitus (T2DM) is a prevalent metabolic disorder that currently affects over 400 million individuals globally and is projected to rise to 552 million by 2030. This condition is linked to a combination of genetic predispositions, lifestyle choices, and environmental influences that contribute to insulin resistance, leading to elevated blood glucose concentrations [1]. T2DM is also a significant risk factor for several debilitating complications, including cardiovascular illnesses, kidney dysfunction, nerve damage, vision loss, and an overall increase in disease incidence and death rates [1, 2]. Insulin release is governed by a meticulous sequence of vesicular transport events that are tightly controlled by membrane transport mechanisms. These include the actions of Rab small GTPases, SNARE proteins, and chaperones associated with SNARE proteins. A decline in insulin secretion could stem from impairments in the transport and maturation of insulin granules, their docking and fusion with the plasma membrane, or abnormal breakdown of insulin and proinsulin [3, 4].

Rab GTPases, which belong to the extensive Ras‐related GTPase superfamily and comprise over 70 distinct members in mammals, play a crucial role in regulating intracellular membrane trafficking and vesicle positioning [5]. These proteins alternate between GTP‐ and GDP‐bound states, functioning as molecular switches to delineate the specific cellular compartments associated with membranes. Certain Rab GTPases are enriched in particular cell types, where they govern distinct membrane transport processes. Rab proteins have been identified as pivotal in insulin secretion in pancreatic beta (β) cells. A study has highlighted a significant link between Rab35 and insulin secretion in these cells [6]. For instance, Rab26 has been identified in the secretory granules of parotid acinar cells, where it regulates the release of amylase [7, 8].

Additionally, Rab26 has been revealed to interact with Rim1, a protein that interacts with Rab3 [9]. A recent study has revealed that Rab26 expression is controlled by the transcription factor MIST1 and is linked to the maturation of secretory granules [10]. Rab34, a small GTPase unique to metazoans, regulates vesicular budding and fusion during exocytosis and endocytosis [11]. Initially discovered in the Golgi complex, Rab34 manages the positioning of lysosomes through its interaction with the Rab‐interacting lysosomal protein (RILP) [11]. Studies have indicated that RILP impedes insulin secretion by enhancing proinsulin degradation and interacting with Rab26, potentially facilitating the fusion of insulin granules with lysosomes [12, 13]. Other research has suggested that Rab26 can limit insulin secretion by sequestering synaptotagmin‐1 [14]. Some Rab proteins are involved in regulating insulin secretion, including Rab37 [15], and Rab2a may mediate the formation of insulin‐secreting bodies. However, Rab27A interacts with motor molecules to regulate the transport of insulin‐secreting bodies. Rab2a and Rab27A interact with Noc2 [16]. Rab3 is similar to Rab27A and participates in the transport and secretion of insulin‐secreting bodies [17]. Currently, the specific role of Rab34 in insulin secretion remains to be elucidated.

MicroRNAs (miRNAs), a class of small non‐coding RNA molecules, are initially transcribed in the nucleus as lengthy primary transcripts. These are then processed by the enzyme Drosha into approximately 70‐nucleotide precursor molecules, known as pre‐miRNAs, which adopt a stem‐loop structure [18]. Subsequently, these pre‐miRNAs are transported to the cytoplasm and further cleaved by the RNase III enzyme Dicer into mature miRNAs, typically around 22 nucleotides in length [18, 19]. A multitude of miRNAs have been implicated in regulating pancreatic β cells and in modulating molecular signaling pathways that are central to the pathogenesis of diabetes [20, 21, 22]. It is well established that miRNAs are crucial in maintaining energy balance, sugar and lipid metabolism, pancreatic β cell development, and insulin secretion [23]. For instance, miR‐720 activates the PI3K/AKT/mTOR signaling pathway, which suppresses insulin secretion [6]. While numerous miRNAs are recognized for their physiological impact on tissues affected by diabetes, the extent of their involvement in diabetes‐related damage remains to be fully elucidated [8]. Research has indicated that miR‐135a modulates insulin signaling and glucose uptake by targeting IRS2 [24]. Additionally, miR‐9 reduces glucose‐stimulated insulin secretion (GSIS) by targeting Onecut2, a transcription factor that suppresses insulin exocytosis [25]. Studies have identified miR‐9 as a negative regulator of insulin secretion from β cells, with the capacity to post‐transcriptionally repress Stxbp1 expression in these cells [26]. The TargetScan database lists over 1200 potential targets of hsa‐miR‐9, including several well‐known oncogenes. Among these, Rab34 has been validated as a direct target of miR‐9 in ovarian carcinoma, as evidenced by Western blotting and dual‐luciferase reporter assays [27]. Furthermore, Luo et al. [28] discovered that miR‐9 is downregulated and may modulate Rab34 in gastric carcinoma. However, the relationship between miR‐9 and Rab34 in insulin secretion remains largely unexplored.

In this study, we investigated the effects of Rab34 on insulin secretion. An increase in Rab34 levels inhibits insulin secretion in β cells. Dual‐luciferase gene reporter assay analysis indicated that miR‐9 is a negative regulator of insulin secretion, targeting Rab34 and restricting insulin secretion in β cells.

## 2 Materials and Methods

### 2.1 Cell Culture and Transfection

INS‐1, Min6, 293A, and 293 T cell lines were bought from the Cell Bank of the Chinese Academy of Sciences (Shanghai, China). 293 T and 293A cell lines were maintained in Dulbecco's modified Eagle medium (DMEM) supplemented with 10% fetal bovine serum (FBS) and 1% penicillin/streptomycin under standard culture conditions (37°C, 5% CO2) and were tested regularly for mycoplasma contamination in the laboratory. Min6 cells were grown in DMEM containing 15% FBS and 55 μM β‐mercaptoethanol. INS‐1 cells were cultured in RPMI 1640 medium supplemented with 15% FBS, 10 mmol/L HEPES, 1 mmol/L PyrNa, and 50 mmol/L β‐mercaptoethanol [1].

### 2.2 Quantitative Real‐Time Polymerase Chain Reaction (PCR)

Total RNA was extracted from β cells using RNAiso Plus (TaKaRa, 9108, CA). The reverse transcription was conducted using 1 μg total RNA, which was used to synthesize cDNA using a PrimeScript RT reagent Kit with gDNA Eraser (TaKaRa, RR047A). Specific Primer sequences used for qPCR have been presented previously [29]. Real‐time quantitative RT‐PCR was performed in a 10 μL reaction volume containing 1 μL cDNA template. The reaction mixture was mixed with cDNA, primers, SYBR, and DNA polymerase following the manufacturer's protocol (SYBR Green I Master from Roche, cat. No. 04913914001). The gene expression of the target mRNA was calculated using the 2^−ΔΔCt^ method. The following real‐time PCR parameters were used for all qPCR reactions: Initial denaturation at 94°C for 30 s, followed by 40 cycles of 5 s denaturation at 94°C, 30 s annealing, and extension at 60°C. All gene expression values were normalized to that of glyceraldehyde‐3‐phosphate dehydrogenase (GAPDH) in the same sample.

### 2.3 CRISPR/Cas9 Mediated Gene Knockout

We reduced Rab34 in INS‐1 cells through the CRISPR/Cas9 technique. The link was CRISPRdirect (dbcls.jp) sgRab34‐1/2 sequences (5‐GGGGCAGCTCCGCCAGGACG‐3 and CCTGGCAGGCGCAAGTGACG) were used for disrupting the Rab34 expression in INS‐1 cells.

### 2.4 pSicoR‐Mediated Gene Silencing and Transfection

Min6 cells were transfected with a recombinant psicor‐shRab34 vector or a control vector to inhibit Rab34 expression. The shRNA sequences targeting Rab34 (mouse species) and a negative control vector (shNC) were constructed. The shRNA sequences are listed in the Table S1. These cells were transfected with a mixture of plasmids and Lipofectamine 2000 (Invitrogen, Carlsbad, CA, USA), according to the manufacturer's instructions. To select stably transfected cells, the culture medium was replaced with a complete medium containing 2 μg/mL puromycin (Thermo Fisher Scientific) after 48 h. The modified expression was confirmed by Western blotting. To evaluate the expression level of miR‐9, cells at 60% confluency were transfected with miR‐9 mimics or an miR‐9 inhibitor.

### 2.5 Construction and Generation of Recombinant Adenovirus Expressing Rab34

Recombinant adenoviruses were generated using AdEasy technology. Recombinant adenoviral genomes were obtained by homologous recombination of shuttle plasmids and the adenoviral backbone plasmid pAdEasy1 [3]. Linearized recombinant adenoviral genomes were transfected into 293A cells to produce recombinant adenoviruses. Transfection efficiency and spread of newly generated recombinant adenoviruses were followed by GFP expression, monitored by fluorescence microscopy. The amplified adenoviruses were titrated and stored at −80°C. The recombinant adenoviruses obtained were designated Ad‐Rab34.

### 2.6 Western Blotting

Proteins were extracted from the cells using a lysis buffer (Beyotime, Haimen, China) containing protease inhibitors. Protein concentrations in the lysates were measured using a BCA kit (Beyotime). Aliquots of protein extracts were subjected to 10%–15% SDS‐PAGE and then transferred to polyvinylidene fluoride membranes (Pall Corporation, New York, NY, USA), which were blocked for 2 h with 5% BSA in tris‐buffered saline, with Tween‐20 (TBST). The membranes were incubated with primary antibodies of Rab34 (1:1000; Proteintech, 27435–1‐AP, USA), proinsulin (1:1000; BioVision, 3106–100, USA), and GAPDH (1:6000; Beijing LABLEAD lnc, AP0063 and 1:5000; Proteintech, 10494–1‐AP, Pearl Street, IL, USA) antibodies. Rabbit polyclonal antibody against insulin (cat. No. 4590 s) was from Cell Signaling Technology. Subsequently, proinsulin monoclonal (mAb) was obtained from HyTest (cat. No. CCI‐17). The mAb against GFP (cat. No. 66002–1‐Ig) was obtained from Proteintech (Wuhan, China). The mAb against synaptotagmin‐1 (Syt1) was acquired from Synaptic Systems (cat. No. 105011). The mAb against Myc (9E10) was obtained from the ATCC (Manassas, VA, USA). Horseradish peroxidase (HRP)‐conjugated secondary antibodies and Texas Red‐conjugated antibodies were obtained from Jackson Immuno Research (cat. No. 111–035‐003; 115–295‐003, West Grove, PA, USA). Following further washing with TBST, blots were visualized using enhanced chemiluminescence reagents (Advansta, Menlo Park, CA, USA). After exposure, protein band densitometry was conducted with the Image Lab software (Bio‐Rad, Hercules, CA, USA).

### 2.7 GST‐Pulldown Assay

The GST‐pulldown assay is generally used to verify in vitro interactions. HEK‐293 T cells were transfected with the indicated plasmids. The cells were then lysed with lysis buffer (containing 20 mM HEPES, pH 7.4, 1% Triton X‐100, 100 mM NaCl, 5 mM MgCl_2_, and EDTA‐free proteinase inhibitor cocktail) for 1 h on ice. The cell lysates were centrifuged at 13,000 × g for 15 min at 4°C. The supernatants were incubated with GST‐fusion protein coupled with GST‐Sepharose 4B resin (GE Healthcare, cat. No. 45–000‐139) at 4°C overnight. GST‐Sepharose 4B resin was washed thrice using the lysis buffer mentioned above but containing different concentrations of NaCl (500, 300, and 100 mM). The bound proteins were analyzed using Western blotting.

### 2.8 Dual‐Luciferase Gene Reporter Assay

Min6 cells were transiently co‐transfected with 1 μg pcDNA3.1‐wild‐type 3′‐untranslated region (UTR) of Rab34 and its mutant region firefly luciferase reporter plasmids, respectively, and 2 ng pRL‐TK luciferase (Promega Corporation, Madison, WI, USA) in the absence or presence of miR‐9. Cells were seeded at a density of 5 × 10^4^ cells/well in a 6‐well plate, transfected using Lipofectamine 2000 (Gibco; Thermo Fisher Scientific Inc.) with the following groups: miR‐9 mimics or NC mimics with PmiR‐WT‐3′UTR Rab34 vector and PmiR‐Mut‐3′UTR Rab34 vector. Following a 48 h transfection, the Firefly and Renilla luciferase activity was measured using the Dual‐Luciferase Reporter Assay System (Promega E1910) following the manufacturer's protocol and normalized to the Renilla luciferase activity.

### 2.9 Confocal Immunofluorescence Microscopy

The cells were fixed for 30 min at room temperature in 4% paraformaldehyde. The cells were then washed thrice with cold PBS for 3 min each and incubated with blocking solution (5% BSA in PBS) for 1 h at room temperature. The cells were permeabilized with 0.1% Triton X‐100 for 15 min and incubated overnight with primary antibodies at 4°C. The cells were rinsed with permeabilization buffer and incubated with fluorescent‐tagged secondary antibodies. The immunolabeled cells were mounted on slides and photographed using a high‐sensitivity laser confocal microscope and a Leica TCS SP8 STED laser scanning confocal microscope.

### 2.10 Enzyme‐Linked Immunosorbent Assay (ELISA) for Insulin Secretion

For insulin secretion in Min6 and INS‐1 cells, cells were pre‐incubated with 100 μL Krebs‐Ringer bicarbonate buffer (KRB) containing 2.8 mM glucose for 60 min. Next, the preincubation buffer was aspirated and replaced with 100 μL of Krebs‐Ringer bicarbonate buffer containing 2.8 mM glucose. At each time point (0–40 min), 2% of the incubation medium was collected for insulin content measurement by mouse insulin ELISA kits (Solarbio, SEKM‐0141, China).

### 2.11 Transmission Electronic Microscopy(TEM)

Min6 cells were infected with Ad‐Vector or Ad‐Rab34. Cells were processed for transmission electron microscopy analysis. The ultrasectioned samples were analyzed using a transmission electron microscope (HT7800 RuliTEM). Morphologically docked granules were defined using the criteria previously described [30]. All granules in β cells were examined, and the cytoplasmic area, granule number, and distance from the plasma membrane were measured using ImageJ software [1].

### 2.12 Statistical Analysis

Data were analyzed using the Statistical Package for the Social Sciences software (version 16.0; SPSS Inc., Chicago, IL, USA), and every experiment was performed thrice. The results are presented as the mean ± standard deviation (SD). Student's *t*‐test was used to compare two independent groups of data. Differences were considered significant at *p* < 0.05.

## 3 Results

### 3.1 Rab34 Knockdown Promoted Insulin Secretion in High Glucose‐Stimulated β Cells

**FIGURE 1:**
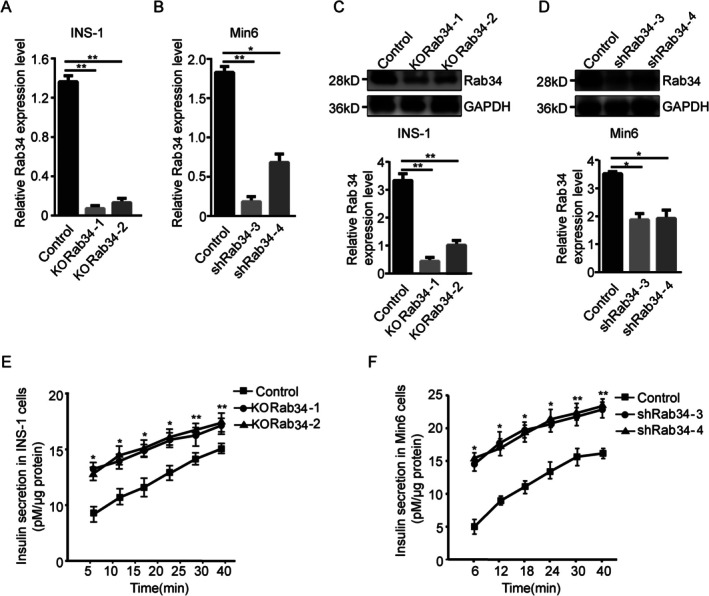
Rab34 knockdown promotes insulin secretion in INS‐1 and Min6 cells. (A–D) RT‐PCR and Western blotting were performed to check the Rab34 knockdown efficiency in INS‐1 and Min6 cell lines. (E‐F) ELISA assay was performed to detect the secretion of insulin in INS‐1 and Min6 cells after Rab34 shRNA‐silencing. The *p*‐values were calculated using a one‐tailed unpaired Student's t‐test. *p < 0.05, **p < 0.01 compared to control groups.

To assess the effects of Rab34 on insulin secretion in β cells, experiments were conducted to examine the efficiency of the knockdown of Rab34 in INS‐1 and Min6 cells. The expression levels of Rab34 from the total protein extraction in INS‐1 and Min6 cells were examined by Western blotting. The mRNA expression levels of Rab34 genes were also examined. The expression levels of Rab34 were decreased in shRNA‐Rab34 cells compared to control cells (Figure 1A–D). Furthermore, insulin secretion was stimulated by 16.7 mmol/L glucose. An ELISA assay was performed to detect the insulin secretion in INS‐1 and Min6 cells, which were depleted of Rab34. The results revealed that Rab34 knockdown significantly promoted insulin secretion (Figure 1E,F).

### 3.2 Rab34 Overexpression Led to Decreased Secretion of Insulin in High‐Glucose‐Stimulated β Cells

**FIGURE 2:**
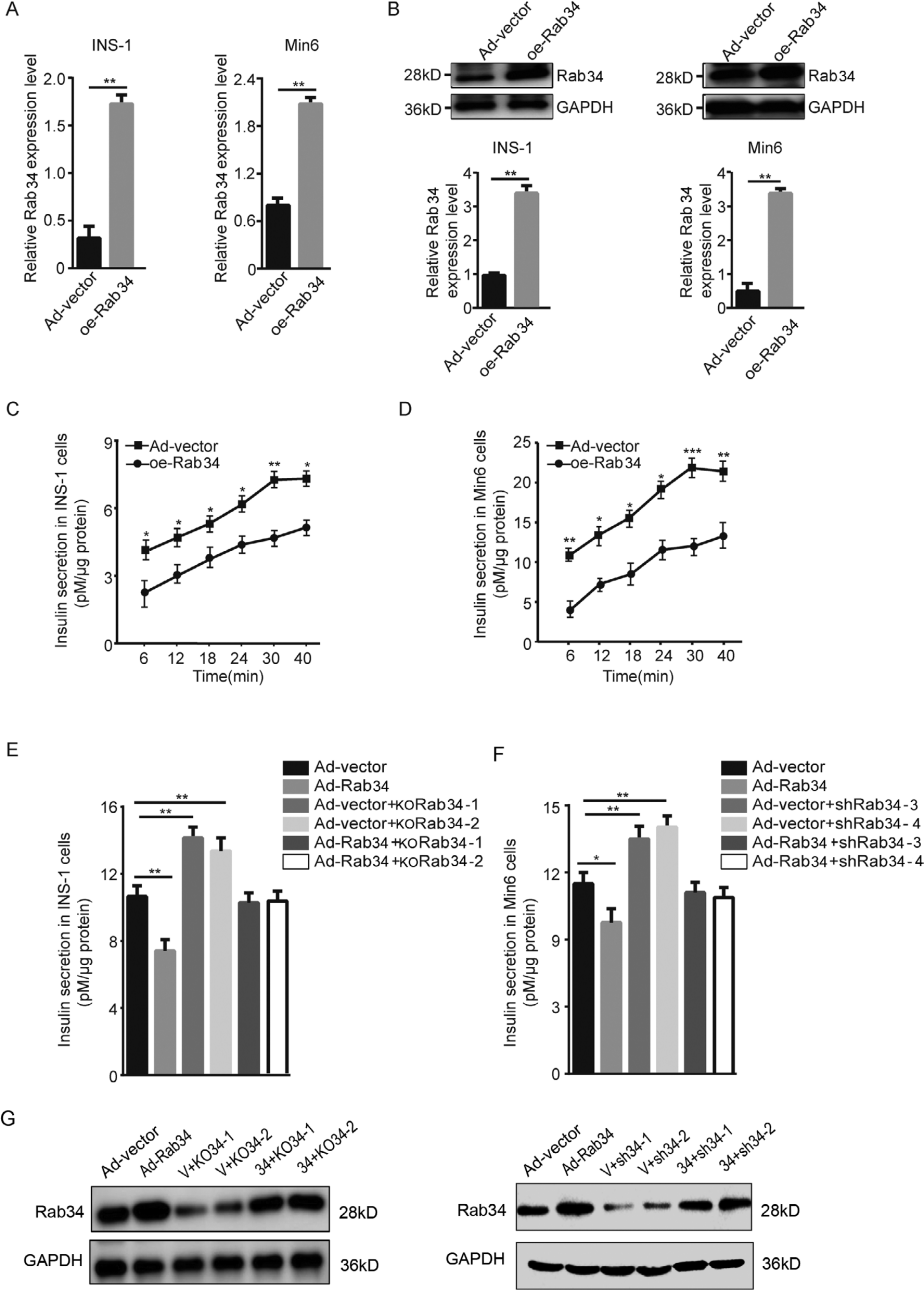
Rab34 overexpression inhibits insulin secretion in β Cells. (B) INS‐1 and Min6 cells were infected with Ad‐Rab34. qPCR and Western blotting were used to detect Rab34 expression. (C‐D) INS‐1 and Min6 cells were infected with Ad‐Rab34. Insulin concentrations were assessed by ELISA. (E‐F) INS‐1 and Min6 cells were infected with Ad‐Rab34 and then transfected with shRab34 to act as rescued experiments. The results are presented as mean ± SD of the expression levels from three independent experiments. *p < 0.05, **p < 0.01 compared to control groups. (G) Western blotting was performed to confirm Rab34a protein expression in rescue experiments.

Next, we examined the effect of adenovirus‐mediated overexpression of endogenous Rab34 on insulin secretion in β cells. The introduction of overexpressed Rab34 through the adenovirus‐mediated expression system markedly and specifically upregulated Rab34 compared with the control groups. As revealed in Figure 2A,B, qPCR and Western blotting assays were used to detect the expression level of Rab34. Insulin secretion was also stimulated by 16.7 mmol/L glucose. Insulin secreted into the media was examined by ELISA. Figure 2C,D reveal that the amount of secreted insulin in INS‐1 and Min6 cells expressing Ad‐Rab34 was significantly decreased. Meanwhile, the above results exhibited that the inhibition of insulin secretion by Rab34 at the 30th min was statistically significant. To further confirm that Rab34 inhibits insulin secretion, rescue experiments were conducted. Moreover, ELISA rescue experiments revealed that forced expression of Rab34 in INS‐1 and Min6 cells resulted in a clear decrease in insulin secretion, and Rab34 knockdown lentiviral infection rescued the decrease in insulin secretion caused by Rab34 overexpression, as presented in Figure 2E,F. Meanwhile, a Western blotting assay was performed to confirm Rab34a protein expression in rescue experiments (Figure 2G). These data further suggested that the normal function of Rab34 was essential for insulin secretion, and Rab34 overexpression inhibited insulin secretion in β cells.

### 3.3 Rab34 Inhibited Insulin Secretion by Promoting Proinsulin Degradation

**FIGURE 3:**
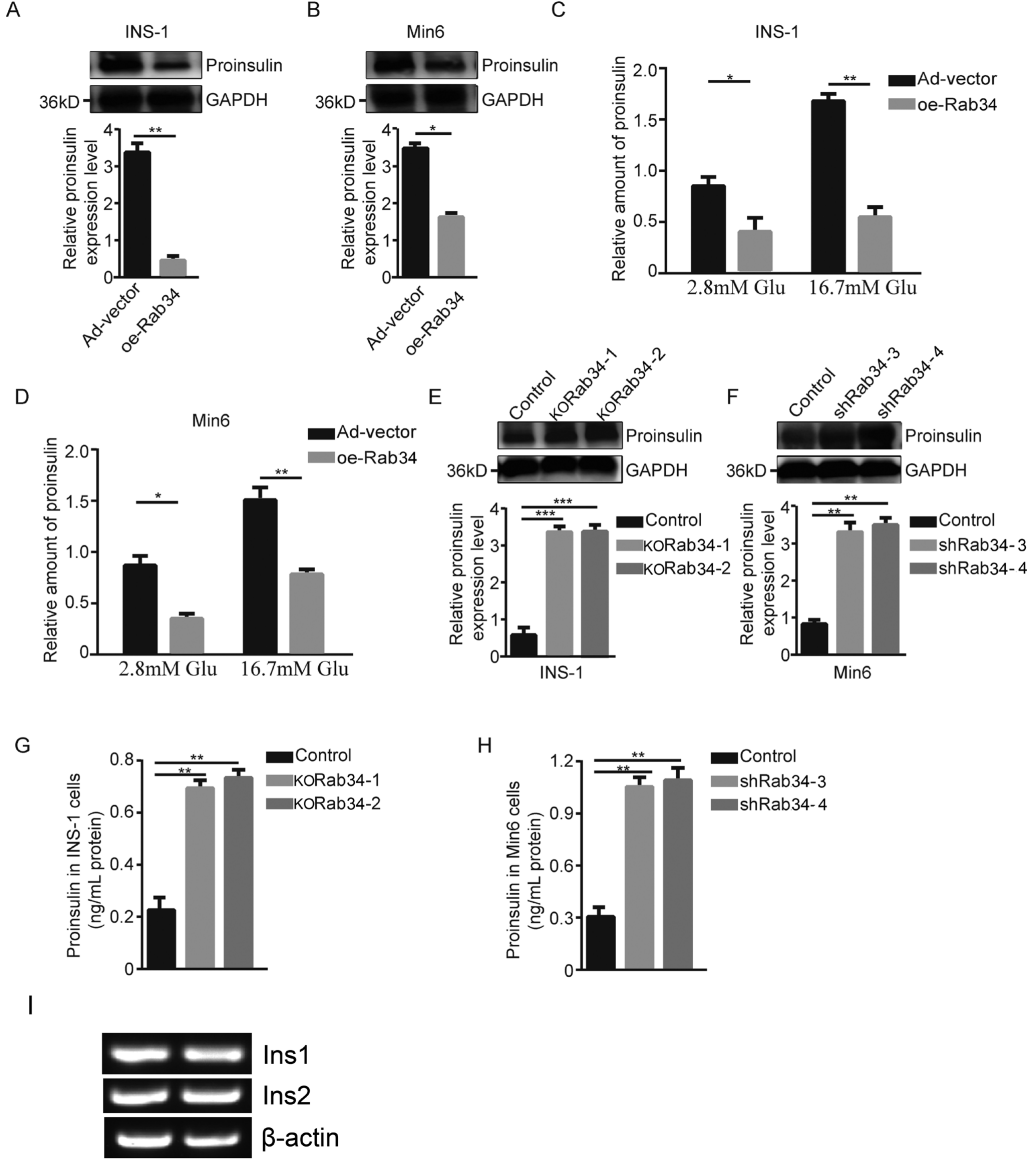
Rab34 inhibits insulin secretion by promoting proinsulin degradation. (A‐B) Western blotting assay was used to detect the protein level of proinsulin in INS‐1 and Min6 cells transfected with Ad‐Rab34. (C‐D) INS‐1 and Min6 cells transfected with Ad‐Rab34 were balanced for 1 h in KRBH buffer containing 2.8 mmol/L glucose and then stimulated with 16.7 mmol/L glucose in KRBH buffer. Intracellular proinsulin was detected by ELISA. (E‐F) Western blotting was used to detect the protein levels of proinsulin in INS‐1 and Min6 cells transfected with sh‐Rab34. The quantitative results from three independent experiments are described in E‐F. (G‐H) Rab34 depletion in INS‐1 and Min6 cells significantly increased the amount of proinsulin detected by ELISA. The results are presented as mean ± SD of the expression levels from three independent experiments. *p < 0.05, **p < 0.01, ***p < 0.001 compared to control groups. (I) mRNA of insulin was unaffected by Rab34.

Proinsulin is the precursor of insulin; therefore, we explored whether Rab34 affects the expression of proinsulin and, thus, insulin secretion. The results revealed that Rab34 could reduce the protein expression level of proinsulin than the control groups in Min6 and INS‐1 cells (Figure 3A,B). An ELISA was used to detect the effect of Rab34 overexpression on proinsulin content under high and low glucose stimulation conditions at the 30th min in Min6 and INS‐1 cells (Figure 3C,D). ELISA results revealed that Rab34 overexpression significantly decreased the proinsulin levels in the high‐glucose stimulation groups (Figure 3C,D). To further demonstrate this, a knockdown experiment was conducted. Western blotting results demonstrated in Figure 3E,F revealed that the protein expression level of proinsulin could increase in pancreatic islet cells when Rab34 was downregulated. ELISA results exhibited that Rab34 downregulation greatly increased the amount of proinsulin (Figure 3G,H). These results indicate that Rab34 alters proinsulin content at the protein level. Is this possible by affecting the transcriptional level of insulin? We transfected Ad‐Rab34 or Ad vector into β cells, extracted mRNA, and detected the transcriptional changes of insulin using RT‐PCR. RT‐PCR revealed that Rab34 overexpression did not affect the transcription levels of insulin genes Ins1 and Ins2, indicating that overexpression of Rab34 does not affect the transcription levels of insulin (Figure 3I). These results collectively indicate a novel function of Rab34 in regulating insulin secretion by promoting proinsulin degradation.

### 3.4 Rab34 Was Associated With Insulin Granules and Decreased the Number of iSGs

**FIGURE 4:**
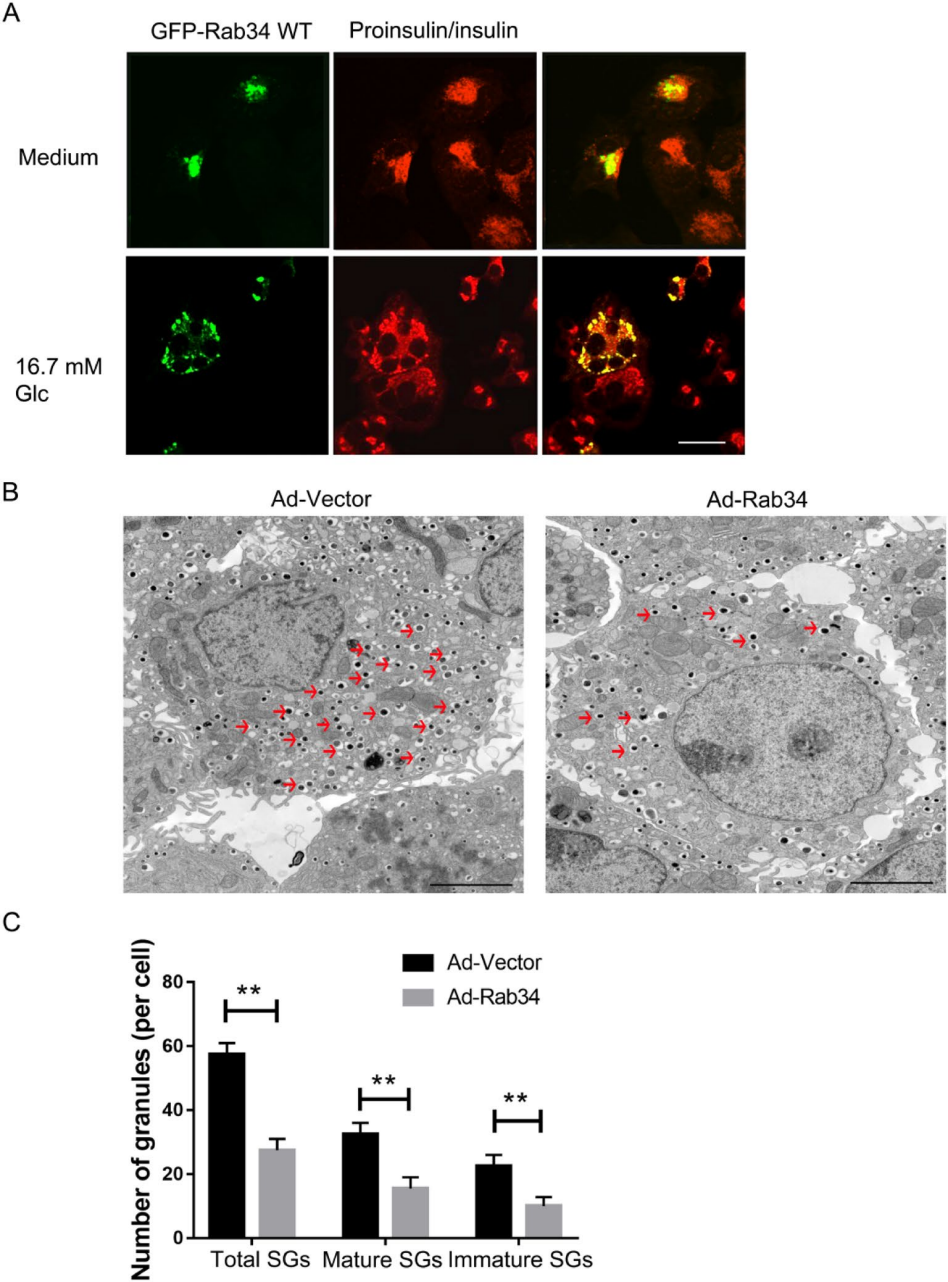
Rab34 induces clustering of insulin granules and decreases the number of iSGs. (A) Min6 cells transfected with GFP‐Rab34 were cultured in a normal medium or KRBH buffer containing 16.7 mmol/L glucose for 40 min and then immunostained with an antibody against insulin/proinsulin, revealing that Rab34 induces insulin granule clustering. (B) TEM images of Min6 cells infected with Ad‐vector or Ad‐Rab34 were observed. (C) Quantitative analysis of mSGs and iSGs demonstrated that Rab34 overexpression resulted in a decreased number of iSGs *n* = 20, *p < 0.05, **p < 0.01.

To detect the effects of Rab34 on the distribution of insulin granules, Min6 cells were transiently transfected with GFP‐Rab34 and immunolabeled with an insulin/proinsulin antibody. Rab34 resulted in the perinuclear clustering of insulin granules in most Min6 cells (> 80%) under normal culture stimulation conditions and a high concentration of glucose (Figure 4A). These results suggest that Rab34 physiologically associates with secretory vesicles and insulin granules and is a regulator of insulin secretion. As proinsulin is an iSG marker, TEM was conducted to prove the decreased number of secretory granules caused by the expression of Rab34. Min6 cells were infected with Ad‐Rab34 or Ad‐vector. The results suggested that the expression of Rab34 decreased the number of insulin iSGs, which probably causes a decrease in insulin release (Figure 4B). The observation from different cell sections (indicated by nuclei of different sizes) revealed that cells expressing Rab34 have fewer secretory granules than the control groups. Further quantitative analysis demonstrated that mature secretory granules (mSGs) and iSGs decreased in cells infected with Ad‐Rab34 (Figure 4C). Collectively, these results suggest that Rab34 causes the retrograde movement of insulin granules to cluster and regulates the biogenesis or degradation of iSGs, thus inhibiting insulin secretion in pancreatic β cells.

### 3.5 Rab34 Induces Insulin Granules Toward Autophagy in Min6 Cells

**FIGURE 5:**
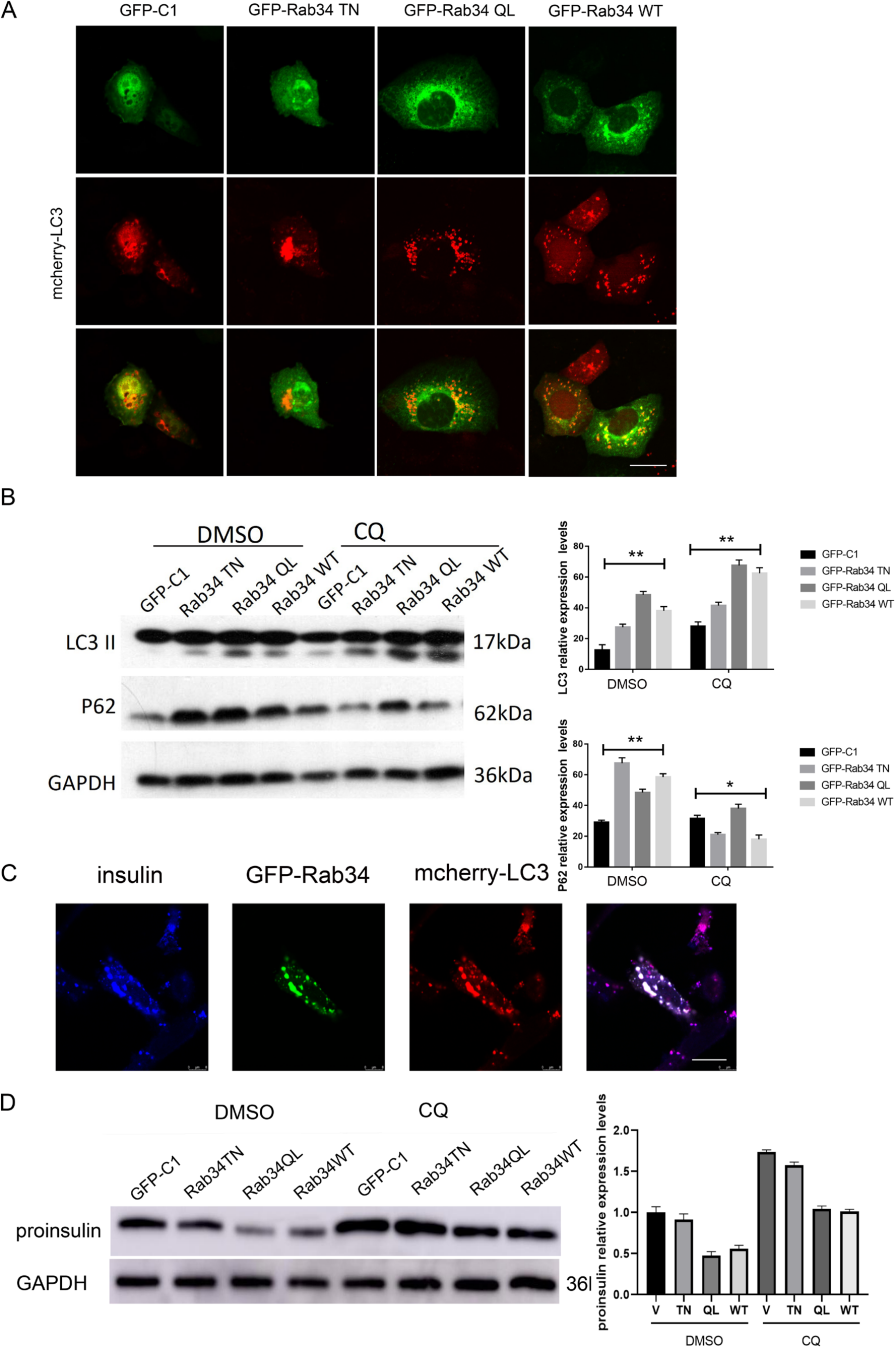
Rab34 induces insulin granules toward autophagy in Min6 cells. (A) GFP‐Rab34 is associated with autophagosomes marked by the LC3 antibody in Min6 cells. Bar = 20 μm. (B) Western blotting revealed that the overexpression of Rab34 WT and Rab34 QL increased the level of LC3II and decreased the level of P62. Quantitative analysis of the results from three independent experiments. *p < 0.05, **p < 0.01. (C) GFP‐Rab34 was associated with insulin at autophagosomes. Bar = 20 μm. (D) Proinsulin protein levels were examined in cells after CQ treatment.

The investigation demonstrated that Rab34 may be engaged in the autophagic pathway. Immunofluorescence microscopy revealed that GFP‐Rab34 was associated with autophagosomes marked by LC3. The results revealed that Rab34 significantly induced autophagosome formation in Min6 cells (Figure 5A). Under normal conditions, overexpression of Rab34WT or Rab34Q111L (active form preferring binding to GTP) mutant induced the formation of LC3 autophagosomes, while the inactivated mutant Rab34T66N (negative form preferring binding to GDP) did not induce the formation of LC3 autophagosomes (Figure 5A). Consistent with the results, Rab34WT and Rab34Q111L but not Rab34T66N induced autophagy. Western blotting demonstrated that Rab34 overexpression increased the protein levels of LC3II under normal conditions and following chloroquine treatment (Figure 5B). When transfected with Rab34 and then immunostained with insulin, Rab34 induced insulin targeting to the autophagosomes marked by LC3 (Figure 5C). To strengthen this hypothesis and further demonstrate Rab34's role in insulin degradation, proinsulin protein levels after CQ treatment were examined in cells overexpressing different Rab34 mutants (Figure 5D).

### 3.6 miR‐9 Regulated Insulin Secretion by Targeting Rab34 in β Cells

**FIGURE 6:**
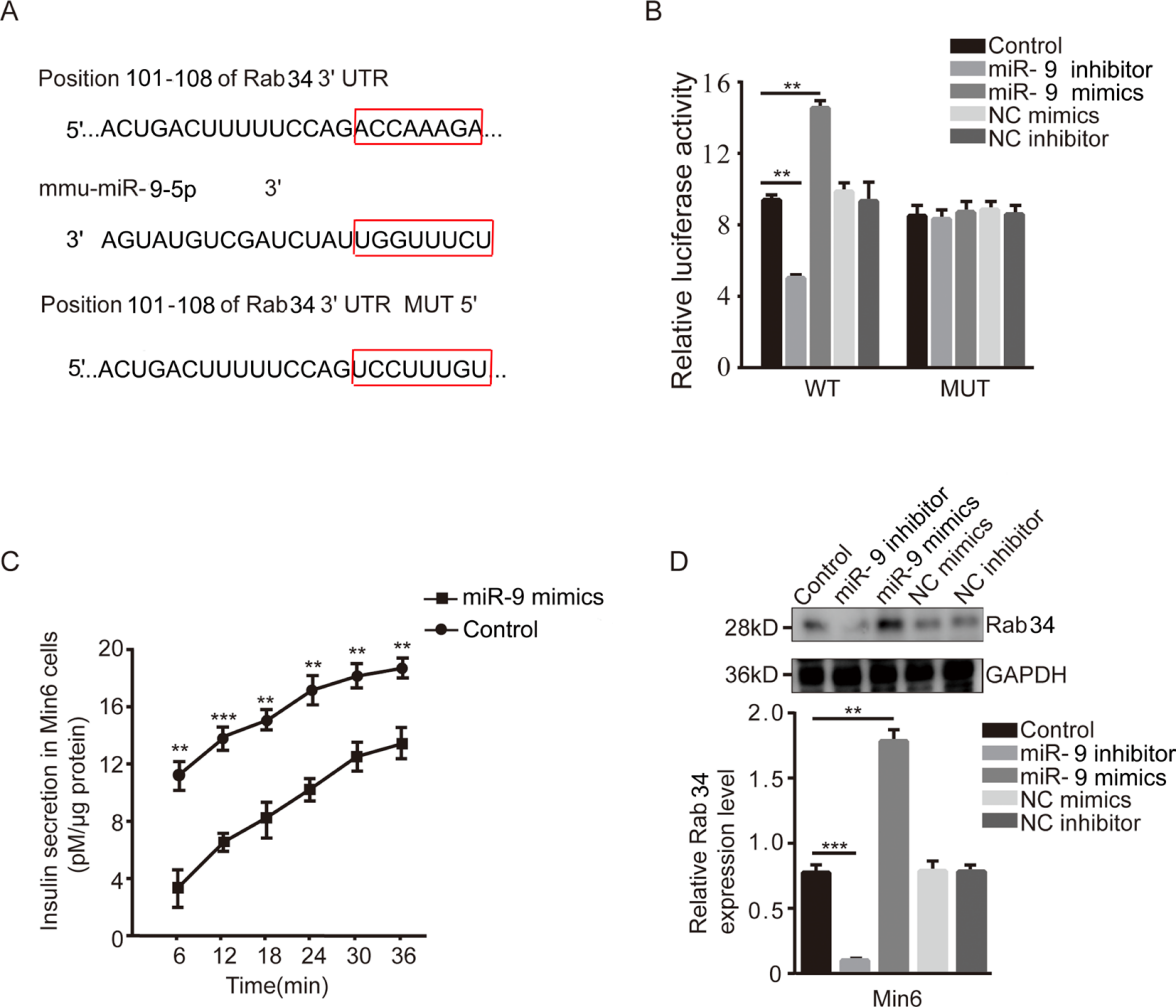
Rab34 is a direct target of miR‐9, which restricts insulin secretion in β cells. (A) The putative miR‐9‐binding sequences in the 3′UTR of Rab34 mRNA were exhibited. A mutation was generated in the Rab34 3′UTR sequence at the complementary site for the seed region of miR‐9. (B) A luciferase assay was performed to explore the effect of miR‐9 on the fluorescence of the recombinant plasmid Rab34 3 UTR sequence. (C) ELISA assay was performed to detect the secretion of insulin in Min6 cells after miR‐9 overexpression. (D) Rab34 expression in Min6 cell lines was detected using Western blotting (up) and RT‐PCR analysis (down) after inhibiting miR‐9 expression. The *p*‐values were calculated using a one‐tailed unpaired Student's t‐test. *p < 0.05, **p < 0.01, ***p < 0.001 compared to control groups.

miRNAs are vital for insulin secretion. One of the important members, miR‐9, has been reported to play an important role in insulin resistance. Meanwhile, we searched for potential upstream target genes of Rab34 using TargetScan. Rab34 was identified as a potential candidate (Figure 6A). To ascertain whether the potential mechanism of miR‐9 affecting insulin secretion was through Rab34, the relationship was based on dual luciferase reporter gene assays. We constructed a recombinant fluorescent expression plasmid of the Rab34 3 UTR sequence, co‐transfected the resulting plasmids with either miR‐9 mimic or scrambled miRNA into Min6 cells, and then tested the effect of miR‐9 on the fluorescence of the recombinant plasmid using a luciferase assay. The results (Figure 6B) revealed that miR‐9 overexpression enhanced the firefly luciferase activity of Rab34 wild type compared with the miR‐NC and miR‐9‐inhibitor groups (*p* < 0.05). Next, we constructed a recombinant fluorescent expression plasmid of the Rab34 3 UTR mutant sequence to avoid the target of miR‐9. However, the data exhibited that there was a non‐significant effect on the firefly luciferase activity of the Rab34 mutant, indicating that functionality depends on the seed sequence of Rab34 mRNA. Next, an ELISA was used to detect the effect of miR‐9 on insulin secretion in Min6 cells. As revealed in Figure 6C, miR‐9 significantly suppressed insulin levels. Insulin secretion was stimulated by 16.7 mmol/L glucose. Moreover, miR‐9 overexpression significantly increased Rab34 expression at the mRNA and protein levels than the control in Min6 cells. Contrarily, miR‐9 downregulation with the miR‐9 inhibitor greatly decreased Rab34 expression (Figure 6D). These results indicated that miR‐9 restricted insulin secretion by targeting to promote Rab34 expression.

## 4 Discussion

Diabetes mellitus, a metabolic disorder, is characterized by elevated blood glucose levels that persist over an extended period. The incidence of this condition has increased globally. Projections from recent studies suggest that the number of individuals with T2DM could reach approximately 439 million by 2030 [1, 30].

The Rab subfamily, which is part of the Ras superfamily of small GTPases, is the most extensive, with over 70 distinct proteins identified in humans [31]. These Rab proteins are key regulators of vesicular transport, overseeing the navigation, docking, and merging of vesicles with their target membranes. Notably, the Rab27 and Rab3 families are highly expressed in cells specialized for secretion and have been extensively studied [32]. Rab34, a Golgi‐resident GTPase, plays a role in lysosome secretion and allocation [11]. It manages the positioning of late endosomes and lysosomes by engaging with RILP [33]. Emerging evidence indicates that Rab34 plays diverse roles in cellular physiology, including its involvement in membrane ruffling and the formation of macropinosomes [34].

Additionally, Rab34 is implicated in regulating protein secretion from the Golgi complex through its interaction with munc13 proteins [35]. Recent findings indicate that RILP influences the lysosomal degradation of proinsulin, which in turn affects the secretion of insulin into the extracellular space, with the involvement of small G proteins such as Rab7 and Rab26 [13, 14]. This suggests a potential role for Rab34 in insulin regulation. Studies have demonstrated that Rab26 can bind directly to the C2A domain of Syt1, thereby inhibiting the release of newly formed insulin granules through exocytosis [14]. Other Rab proteins, such as Rab35, are also involved in modulating insulin secretion [36], underscoring the significance of the Rab family in the study of insulin secretion and diabetes.

miRNAs are crucial regulators in insulin secretion, pancreatic development, and β cell differentiation. Numerous miRNAs have been associated with diabetes and associated conditions, with positive and negative correlations identified. While aberrant miRNA expression patterns are known to be correlated with diabetes and the regulation of insulin secretion, the specific functions and impact of these miRNAs remain incompletely comprehended. miR‐9 has been implicated in various pathological conditions, including various cancers, neurological disorders, and inflammatory diseases [37, 38]. Packer et al. [38], revealed that miR‐9 is involved in Huntington's disease. Research has indicated that miR‐9 can reduce GSIS by targeting the transcription factor Onecut2, thereby suppressing granuphilin expression, which is a negative regulator of insulin exocytosis [25]. Hu, D et al. [26] have also recognized miR‐9 as a significant modulator of insulin secretion in β cells. Studies utilizing pancreatic islet cell lines have demonstrated that elevated miR‐9 levels lead to decreased GSIS, whereas reduced miR‐9 levels can enhance insulin secretion. Additionally, it has been observed that miR‐9 and Stxbp1 are essential for the proper functioning of β cells. However, the interaction between miR‐9 and Rab proteins, which are also involved in insulin secretion, remains to be investigated.

In this study, we investigated the role of miR‐9 in regulating insulin release and its interaction with Rab34. Our findings revealed that Rab34 plays a dual role in pancreatic β cells by suppressing insulin secretion and simultaneously facilitating the breakdown of proinsulin. Furthermore, our study demonstrated that miR‐9 influences insulin secretion by directly targeting Rab34. Insulin, a key hormone that regulates glucose metabolism, is crucial for maintaining glucose homeostasis through proper secretion. Both domestic and international studies have established that miRNAs, including miR‐9, are involved in modulating insulin secretion [39, 40]. This study contributes to the growing body of knowledge on the impact of miRNAs in this process.

**FIGURE 7:**
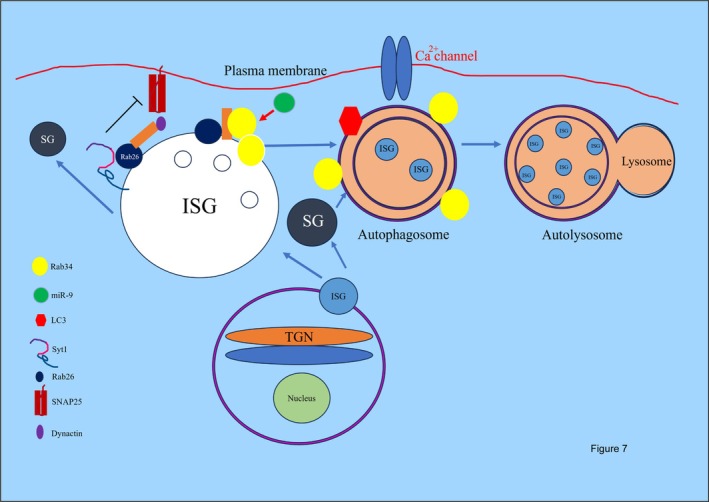
A model proposes miR‐9 restricts insulin secretion by targeting Rab34. Which mediates the lysosomal degradation of proinsulin.

In summary, we propose that miR‐9 triggers Rab34 expression, which in turn limits insulin secretion from pancreatic β cells. We introduced a model wherein miR‐9 curbs insulin secretion by directing Rab34 to mediate the lysosomal degradation of proinsulin. Rab34 inhibits the forward transport of insulin particles by aggregating them around the nucleus. In cells overexpressing Rab34, the numbers of mature and immature insulin granules decreased. Rab34 mediates the reverse transport of insulin particles and participates in the formation or degradation of immature insulin particles, thereby inhibiting the extracellular secretion of insulin in β cells. According to this model, Rab34 attracts insulin granules and engages LC3 to form autophagosomal membranes (Figure 7).

In this model, Rab34 recruits insulin granules and subsequent LC3 to the autophagosomal isolated membrane.

## Funding

This work was supported by Hunan Provincial Natural Science Foundation of China, 2023JJ50467. Scientific Research Project of Hunan Provincial Department of Education, 24A0636.

## Conflicts of Interest

The authors declare no conflicts of interest.

## Supporting information


**Figure S1:** Significant upregulation of Rab34 expression in β‐cells under high glucose and palmitic acid induced stress. A Western blot showed that Rab34 protein expression increased after 25 mM glucose stimulation for different time in INS‐1 cells. B Quantitative analysis of the results of A from 3 independent experiments. C To test the dose‐dependent effect of PA on INS‐1 cells, cells were treated with 0, 40, 80, 120, and 160 μM PA for 48 h. Western blot showed that Rab34 protein expression increased after PA stimulation for different concentration. D Quantitative analysis of the results of C from 3 independent experiments (***p < 0.001, **p < 0.01, *p < 0.05, t tests. PA, palmitic acid). E, F RT‐PCR was performed to detect the Rab34 mRNA expression levels under high glucose and palmitic acid induced stress in INS‐1 cells. The *p* values were calculated using one‐tailed unpaired Student's *t* test (* *p* < 0.05, **p < 0.01, ***p < 0.001 compared to control groups).

**Table S1:** Oligos used in experiments.

## Data Availability

The data that support the findings of this study are available on request from the corresponding author. The data are not publicly available due to privacy or ethical restrictions.
