# Supplementary material for: miR‐9 Restricts Insulin Secretion by Targeting Rab34, Which Mediates Lysosomal Degradation of Proinsulin
Source: Kaohsiung J Med Sci. 2026 Mar 27;42(10):e70202. doi: 10.1002/kjm2.70202 (PMC13399614; doi:10.1002/kjm2.70202)
Supplement: Supplementary file 1 [file KJM2-42-e70202-s001.docx]

**Supplementary information**

**miR-9 restricts insulin secretion by targeting Rab34 which mediates lysosomal degradation of proinsulin**

Supplementary Figure 1 legend

**Supplementary Fig.1 Significant upregulation of Rab34 expression in β-cells** **under high glucose and palmitic acid induced stress.** A Western blot showed that Rab34 protein expression increased after 25 mM glucose stimulation for different time in INS-1 cells. B Quantitative analysis of the results of A from 3 independent experiments. C To test the dose-dependent effect of PA on INS-1 cells, cells were treated with 0, 40, 80, 120, and 160 μM PA for 48 h. Western blot showed that Rab34 protein expression increased after PA stimulation for different concentration. D Quantitative analysis of the results of C from 3 independent experiments. (***P < 0.001, **P < 0.01, *P < 0.05, t tests. PA, palmitic acid). E, F RT-PCR was performed to detect the Rab34 mRNA expression levels under high glucose and palmitic acid induced stress in INS-1 cells. The p values were calculated using one-tailed unpaired Student’s t test. (* p<0.05, **p < 0.01, ***p < 0.001 compared to control groups)
