# Supplementary material for: miR‐9 Restricts Insulin Secretion by Targeting Rab34, Which Mediates Lysosomal Degradation of Proinsulin
Source: Kaohsiung J Med Sci. 2026 Mar 27;42(10):e70202. doi: 10.1002/kjm2.70202 (PMC13399614; doi:10.1002/kjm2.70202)
Supplement: Supplementary file 2 [file KJM2-42-e70202-s002.docx]

Supplement Table 1

Oligos used in experiments

Targeting sequences for shRNA

| shRab34-3# | 5’ CGGCACATTGCAGATGTTGTT 3’ |
| --- | --- |
| shRab34-4# | 5’ GGCTACCATCGGAGTGGATTT 3’ |
