# Supplementary material for: miR‐9 Restricts Insulin Secretion by Targeting Rab34, Which Mediates Lysosomal Degradation of Proinsulin
Source: Kaohsiung J Med Sci. 2026 Mar 27:e70202. Online ahead of print. doi: 10.1002/kjm2.70202 (PMC13399614; doi:10.1002/kjm2.70202)
Supplement: Supplementary file 1 — Figure S1: Significant upregulation of Rab34 expression in β‐cells under high glucose and palmitic acid induced stress. A Western blot showed that Rab34 protein expression increased after 25 mM glucose stimulation for different time in INS‐1 cells. B Quantitative analysis of the results of A from 3 independent experiments. C To test the dose‐dependent effect of PA on INS‐1 cells, cells were treated with 0, 40, 80, 120, and 160 μM PA for 48 h. Western blot showed that Rab34 protein expression increased after PA stimulation for different concentration. D Quantitative analysis of the results of C from 3 independent experiments (***p < 0.001, **p < 0.01, *p < 0.05, t tests. PA, palmitic acid). E, F RT‐PCR was performed to detect the Rab34 mRNA expression levels under high glucose and palmitic acid induced stress in INS‐1 cells. The p values were calculated using one‐tailed unpaired Student's t test (* p < 0.05, **p < 0.01, ***p < 0.001 compared to control groups). [file KJM2-9999-e70202-s002.docx]

**Supplementary information**

**miR-9 restricts insulin secretion by targeting Rab34 which mediates lysosomal degradation of proinsulin**

**
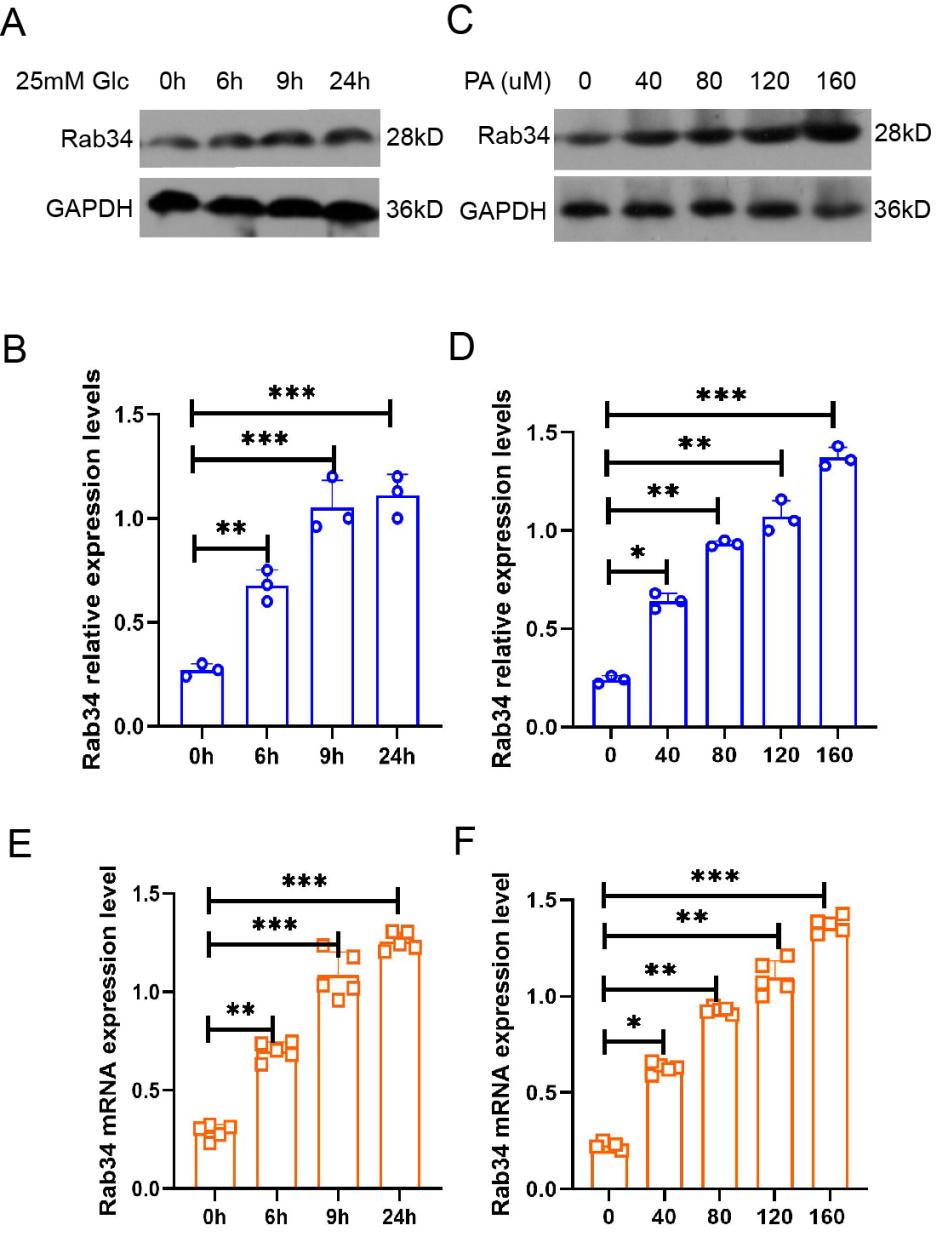
**

Supplementary Figure 1 legend

**Supplementary Fig.1 Significant upregulation of Rab34 expression in β-cells** **under high glucose and palmitic acid induced stress.** A Western blot showed that Rab34 protein expression increased after 25 mM glucose stimulation for different time in INS-1 cells. B Quantitative analysis of the results of A from 3 independent experiments. C To test the dose-dependent effect of PA on INS-1 cells, cells were treated with 0, 40, 80, 120, and 160 μM PA for 48 h. Western blot showed that Rab34 protein expression increased after PA stimulation for different concentration. D Quantitative analysis of the results of C from 3 independent experiments. (***P < 0.001, **P < 0.01, *P < 0.05, t tests. PA, palmitic acid). E, F RT-PCR was performed to detect the Rab34 mRNA expression levels under high glucose and palmitic acid induced stress in INS-1 cells. The p values were calculated using one-tailed unpaired Student’s t test. (* p<0.05, **p < 0.01, ***p < 0.001 compared to control groups)
