# Supplementary material for: miR‐9 Restricts Insulin Secretion by Targeting Rab34, Which Mediates Lysosomal Degradation of Proinsulin
Source: Kaohsiung J Med Sci. 2026 Mar 27:e70202. Online ahead of print. doi: 10.1002/kjm2.70202 (PMC13399614; doi:10.1002/kjm2.70202)
Supplement: Supplementary file 2 — Table S1: Oligos used in experiments. [file KJM2-9999-e70202-s001.docx]

Supplement Table 1

Oligos used in experiments

|  | \| Targeting sequences for shRNA \|  \|  \| \| \| --- \| --- \| --- \| --- \| \| \| shRab34-3# \| 5’ CGGCACATTGCAGATGTTGTT 3’ \| \| --- \| --- \| \| shRab34-4# \| 5’ GGCTACCATCGGAGTGGATTT 3’ \| \| \| \| |
| --- | --- | --- | --- | --- | --- | --- | --- | --- | --- | --- | --- | --- |
